# Evidence of sensory error threshold in triggering locomotor adaptations in humans

**DOI:** 10.1371/journal.pone.0321949

**Published:** 2025-04-29

**Authors:** Emily M. Herrick, Sergiy Yakovenko

**Affiliations:** 1 Department of Chemical and Biomedical Engineering, West Virginia University, Morgantown, WV, United States of America; 2 Division of Exercise Physiology, Department of Human Performance, West Virginia University, Morgantown, WV, United States of America; 3 Department of Neuroscience, West Virginia University, Morgantown, WV, United States of America; 4 Department of Mechanical and Aerospace Engineering, West Virginia University, Morgantown, WV, United States of America; 5 Rockefeller Neuroscience Institute, West Virginia University, Morgantown, WV, United States of America; Fondazione Santa Lucia Istituto di Ricovero e Cura a Carattere Scientifico, ITALY

## Abstract

Changing body biomechanics or external conditions trigger neural adaptations to optimize motor behavior. While the adaptations appear to be constantly minimizing movement errors, not all errors necessarily initiate sensorimotor adaptations. The locomotor control system may resist changes since exploratory modifications can lead to critical failures in walking. Theoretically, this implies the presence of an error threshold to trigger the adaptation mechanism. Here, kinematic and kinetic asymmetries were imposed as conditions on stepping using a passive orthosis (kinematic asymmetry) and real-time feedback about limb loading (kinetic asymmetry) to vary sensorimotor error during locomotion on a treadmill. Healthy participants adapted to asymmetric conditions while walking on a tied-belt treadmill. The asymmetry in leading and trailing double stance captured the presence of aftereffects, and consequently adaptation, in two conditions: i) only kinematic constraints, or ii) kinematic and kinetic constraints. We tested the hypothesis that the presence of adaptation depends on the magnitude of locomotor asymmetry. Kinematic asymmetry alone did not induce persistent locomotor adaptation; however, the addition of asymmetric interlimb loading triggered the expected adaptation. This result suggests that uninjured locomotor systems can cope with a range of kinematic asymmetries without initiating persistent adaptations, and that loading may be a key variable for triggering the adaptation. The error threshold within the adaptation mechanism may mitigate possible disruption of locomotion when adaptation is not necessary. These insights elucidate the mechanism of neural plasticity and have implications for rehabilitation.

## Introduction

Human walking is a versatile behavior that adapts not only to unpredictable environments but also to errors within neural and musculoskeletal dynamics, for example, due to neurological or peripheral changes [1]. Since the locomotor function is vital for all terrestrial vertebrates, enabling navigation to find food, evade predators, and procreate [2,3], the adaptation of the locomotor system to body changes and surroundings [4,5] may be contrasted by the danger of maladaptations, when exploratory adjustments of this fundamental motor behavior may lead to critical failures and reduced biological fitness. However, the locomotor system is robust [6] and hierarchically organized [3,7], allowing it to cope with errors in planning and execution using multiple mechanisms [1]. The difference between planned and executed movement and posture is minimized by viscoelastic muscle responses, also known as pre-flexes, sensory feedback actions, and predictive feedforward drive [3,8]. The feedback mechanisms can rapidly compensate for minor and unpredictable external perturbations, for example, using the short-latency adjustments to the ongoing activity and also through the use-dependent Hebbian style learning within these pathways [9]. Yet, the neural pathways involved in pattern generation and coordination may be required to change when body configuration or demands change, for example, due to aging, trauma, or object interactions. Repeated exposure to a novel mechanical constraint that causes error feedback can engage modifications and gradually optimize movement to new motor demands. The removal of the constraint often reveals aftereffects and requires de-adaptation to return the system to the previous optimal state, as illustrated in Helmholtz’s prism adaptation task [10–13]. Similar to other motor pathways, the locomotor system has been shown to exhibit adaptation and de-adaptation processes, for example, in walking on a split-belt treadmill with asymmetric stepping [14–17]. Multiple pathways within the motor hierarchy may be required to explain abrupt and gradual locomotor adaptations, as memories are created, recalled or even unlearnt [18].

**Fig 1:**
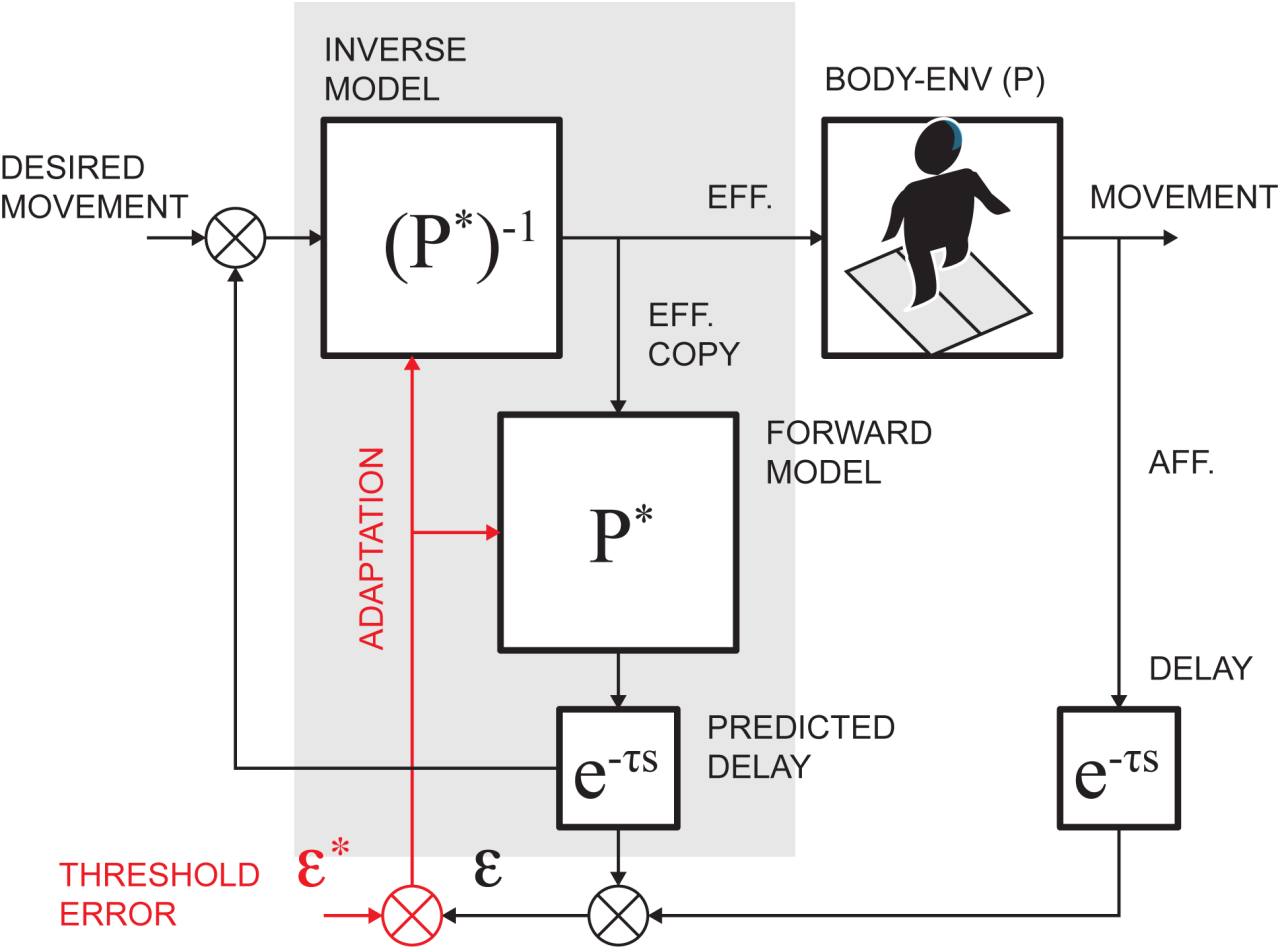
Schematic of the internal dynamic representations for movement execution. P and P* denote body-environment interaction dynamics and its embedded representation, respectively. The embedded dynamics in the shaded area can be used for planning and monitoring of movement execution. The hypothetical adaptation pathway (red) may cause changes in dynamic transformations to reduce discrepancy in predicted and observed sensory errors when this difference exceeds a threshold error (ε*).

The prominent computational framework explaining closed-loop movement control is based on the internal models of body dynamics [19] used in movement planning and execution. The inverse models of body dynamics, see the (P*)^-1^ element in Fig 1, can transform kinematic signals into motor commands (*efference*). To overcome processing and transmission delays, the collateral feedforward motor signal (*efference copy*) is processed through the internal forward model (P*) to predict and compare sensory feedback during movement execution, as shown in Fig 1. The *external* errors can then be used within the inverse models to generate the automatic compensation to the ongoing motor command. The mismatch (ε) of expected and actual sensory signals identifies unexpected *external* or *internal* errors. The *internal* error may require adaptation of the execution and prediction pathways to reduce the discrepancy following a movement. In the process of motor adaptation, sensory errors drive the process, resolving the sensorimotor predictions through the internal representation in the cerebellum, as was shown in reaching tasks [11]. The recalibration of the internal model was generally implicated in motor tasks [20] and specifically in the split-belt locomotor studies with large limb speed differences [21,22]. While adaptation of the internal model can optimize body movement in changing conditions, reducing overall effort [23,24], there is likely a cost associated with the adaptation and de-adaptation processes. Thus, it is likely that the locomotor system may initiate adaptation only after perceived error exceeds a putative error threshold (ε*), demarcating errors tolerated by the control system and those initiating adaptation within internal representations.

In this study, we examined the mechanism of locomotor adaptations during asymmetric gait, which has been previously studied in the context of developing effective rehabilitation for individuals with neurological conditions caused by stroke [22,25,26] and other neurological conditions, for example, parkinsonism [27–30]. Split-belt treadmills were used to impose asymmetrical walking using large speed differences that can trigger the adaptation process and transfer aftereffects to overground walking [21]. However, small limb speed differences are behaviorally related to turning, as shown by experimental studies of walking on a curve [31,32] and simulations [33], and not to the limb preference typically seen in gait pathologies [34]. For this reason, we used kinematic and kinetic constraints during tied-belt locomotion that were expected to cause gait asymmetry and potentially trigger locomotor adaptation. We hypothesized that there is a threshold mechanism that limits the initiation of locomotor adaptation in response to asymmetric errors, which would be evident from persistent aftereffects. Persistent aftereffects were expected in response to kinematic and kinetic interlimb asymmetries but not to kinematic asymmetry alone.

## Materials and methods

### Description of population, instrumentation, and experimental procedures

All procedures were approved by West Virginia University Institutional Review Board for the period of April 14, 2021 to April 13, 2022. All participants provided written informed consent. To test the presence of a sensory error threshold, the amount of imposed sensory error was varied by having one group that experienced low sensory error and another group that experienced high sensory error. If the high sensory error group demonstrated aftereffects of the imposed asymmetry while the low sensory error group did not demonstrate similar aftereffects, then it was determined that (i) the sensory error threshold exists, and (ii) the high sensory error group experienced enough sensory error to cross the said threshold and engage the adaptation mechanism while the low sensory error group did not.

**Fig 2:**
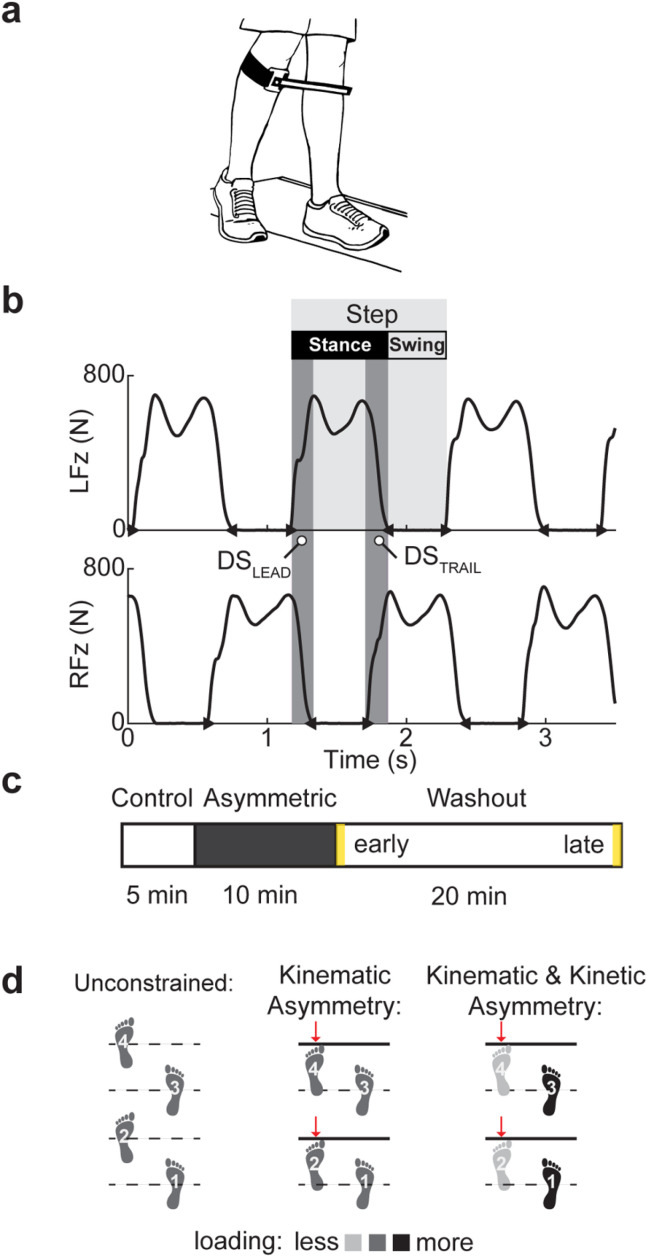
Methodological setup of instrumentation and composition of a testing session. (a) Subject instrumentation with a kinematic constraint. (b) Example of processed ground reaction forces with the detected events defining swing, stance, and lead/trail double stance subphases (DS_LEAD_, DS_TRAIL_). (c) The session timeline was with early and late washout blocks of 20 steps in yellow. (d) Diagram of locomotor conditions: unconstrained, kinematic-only asymmetry, kinematic and kinetic asymmetry. Treadmill belt speed was set to 1.0 m/s in all conditions for both legs.

As such, 23 healthy adults (25.0 ± 6.2 y, 12 males, 11 females) with no known neurological disease or persistent musculoskeletal injury consented to participate in the experiment. The participants were divided into two groups: (i) Group 1 (N=11, 24.5 ± 3.5 y, 4 males, 7 females), the group designated to perform the walking task with only a kinematic asymmetry and thus experience low sensory error, and (ii) Group 2 (N=12, 25.4 ± 8.1 y, 8 males, 4 females), the group designated to perform the walking task with simultaneous kinematic and kinetic asymmetries and thus experience high sensory error. All conditions were tested on a treadmill instrumented with ground reaction force sensors (Bertec, Columbus, OH). The kinematic asymmetry was imposed using a unilateral stride constraint, limiting the swing of the affected limb during walking (Fig 2). The kinematic constraint was secured on the right limb with a strap fastened below the knee, protruding anteriorly 2 cm (for Group 1) and 10.5 cm (for Group 2) and medially by 25 cm, constraining the left limb excursion. The adjustment in anterior protrusion between groups allowed participants to swing the constrained leg several centimeters beyond the placement of the contralateral leg, decreasing the probability that they would adopt the strategy of walking with faster steps. While walking with this device, participants were instructed to prevent contact with the bar. The kinetic asymmetry was imposed by explicitly instructing the participant prior to the data collection to shift the loading of the constrained (left) leg during walking, mimicking limping. The experimenter monitored the vertical component of ground reaction force for each limb via real-time plotting of the signals, and verbal feedback was given to the participant if the peak ground reaction force and stance time for the right leg were not about 10% greater than that for the left leg.

Each session consisted of three trials: control, adaptation (i.e., *Asymmetric*), and washout, see Fig 2c. During all trials, the treadmill was set in a tied-belt configuration with each belt moving at 1 m/s. The control trial documented the participant’s baseline gait pattern for 5 minutes. During the asymmetric trial, the participants walked with the constraint(s) designated by their group for 10 minutes. The washout period of 20 minutes allowed the observation of aftereffects after the removal of the constraints. All trials were done sequentially without the participant resting in between the trials to avoid washing out any aftereffects prior to the washout trial.

### Data processing

#### Ground reaction forces and events.

Ground reaction forces were calibrated and sampled at 1 kHz (National Instruments, Austin, TX). The onsets and offsets of each stance period were detected using a supervised automated thresholding method from the raw vertical force components [35]. Step cycle phases were analyzed based on the differences between consecutive events, as illustrated in Fig 2b. The leading and trailing *double stance* sub-phases were defined as the periods when both limbs were on the ground in stance (denoted as ${DS}_{LEAD},\;{DS}_{TRAIL}$ in Fig 2d, respectively). A subset of the automatically detected events was visually inspected, and all data was treated with an additional standard outlier rejection method to remove any minor inaccuracies in event detection [36]. The leading and trailing double stance periods were smoothed with a moving average (sliding window length, k = 20).

#### Limb loading.

To quantify the loading afforded to each limb in each condition, we evaluated the cumulative sum of vertical ground reaction forces during the stance phase of the gait cycle. The cumulative sums were normalized to the participant’s weight and the number of steps in each condition.

#### Loading analysis.

The purpose of the loading analysis was to verify that Group 1 had only asymmetric kinematics and symmetric limb loading, while Group 2 had asymmetric limb loading, indicating the presence of kinematic and kinetic asymmetries. This dissociated kinematic only and kinematic-kinetic conditions in our study. The loading analysis served as a confirmation that the expected conditions for each group were present before testing the main hypothesis that Group 2 would have aftereffects present in the washout condition while Group 1 would not show the same aftereffects (see Aftereffects Analysis). A Kolmogorov-Smirnov test indicated that the loading values for the left and right limbs were normally distributed for each group. As such, the difference in limb loading was tested using a paired, two-tailed t-test for Group 1 and a paired, left-tailed t-test for Group 2. The level of significance was α = 0.05 in all statistical tests.

#### Asymmetry index.

To quantify temporal asymmetry in all conditions, we calculated an asymmetry index (AI) based on the double stance subphases for each step. The AI was calculated as the absolute value of the ratio between the difference and sum of *leading* and *trailing* double stance periods (see ${DS}_{LEAD},\;{DS}_{TRAIL}$ in Fig 2b):


$$AI=\left|\frac{{DS}_{LEAD}-\;{DS}_{TRAIL}}{{DS}_{LEAD}+\;{DS}_{TRAIL}}\right|$$ (1)

The deviations from zero indicated lateralized gait asymmetry. A temporal asymmetry, as opposed to a spatial asymmetry, was chosen to track adaptation because we studied locomotion on a tied-belt treadmill, where both legs moved at the same speed. The stride length, defined as the distance between successive heel strikes of the same foot, was expected to fluctuate minimally due to the step-to-step variability of speed. However, we expected no consistent interlimb stride length changes in the asymmetric tasks because both limbs covered the same distance over the same period. As expected, the step constraint was observed to change the relative timing of phases in the preliminary analysis.

#### Aftereffects analysis.

To test the existence of aftereffects, we compared the participant’s temporal asymmetry in the early and late portions of the washout condition. We expected that the asymmetry would decrease throughout the washout condition, which will be evident from comparing the absolute value of the AI for the first and last 20 steps of the washout trial (denoted as early and late washout, respectively), similar conceptually to the comparison of early and late adaptation [37]. The directionality of any asymmetry was not considered, for only the magnitude of the asymmetry was relevant to our analysis. A Kolmogorov-Smirnov test indicated that the AI values for the early and late washout periods were normally distributed for each group. As such, the absolute difference in AI was tested between early and late washout periods using a paired, two-tailed t-test for Group 1 and a paired, right-tailed t-test for Group 2 (α = 0.05).

## Results

**Fig 3:**
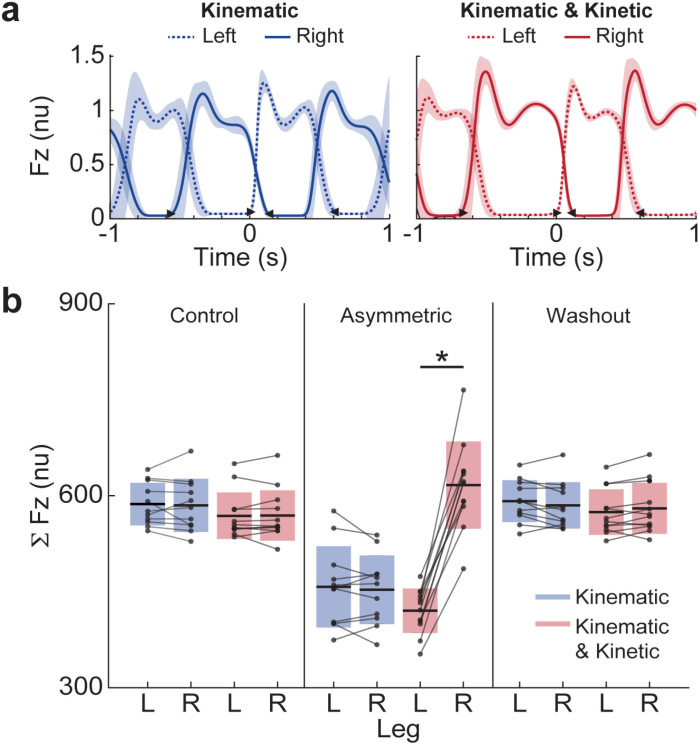
Analysis of loading symmetry in Groups 1 (*kinematic* constraint only) and 2 (*kinematic* and *kinetic* constraints). (a) Examples of vertical ground reaction force profiles (low-pass filtered, cutoff frequency at 100 Hz) for a representative participant. Means with the standard deviations of forces were normalized to body weight and aligned on the onset of stance. (b) The group analysis of limb loading is shown with the comparison of mean (± standard deviation) cumulative sum of the vertical force in all conditions. The units were normalized (nu) to body weight and the number of steps in each condition. The thin lines across the box plots show individual participant data. * Indicates a significant difference with a p < 0.05.

We found that the asymmetric loading, in addition to the kinematic asymmetry, was needed for the development of aftereffects. Fig 3 compares the loading between the left and right limbs in each condition for each group. The limb loading (cumulative sum of vertical forces) was normally distributed in each leg across groups and conditions (**Control:** Group 1: left limb, p = 0.82, right limb, p = 0.93, Group 2: left limb, p = 0.33, right limb, p = 0.41; **Asymmetric:** Group 1: left limb, p = 0.86, right limb, p = 0.96, Group 2: left limb, p = 0.95, right limb, p = 0.61; **Washout:** Group 1: left limb, p = 0.99, right limb, p = 0.78, Group 2: left limb, p = 0.71, right limb, p = 0.78). The means and standard deviations (SD) are reported below. In the asymmetric condition, there was no significant difference in loading values between the left and right limbs in Group 1 (loading values were similar for left limb = 458 (±64) and for right limb = 453 (±54), p = 0.53); however, there was a significant difference in Group 2 (loading values for left limb = 420 (±35) and for right limb = 617 (±68), p < 0.001). This indicates that the additional kinetic condition was successfully executed, and the kinematic constraint alone did not promote differences in loading.

**Fig 4:**
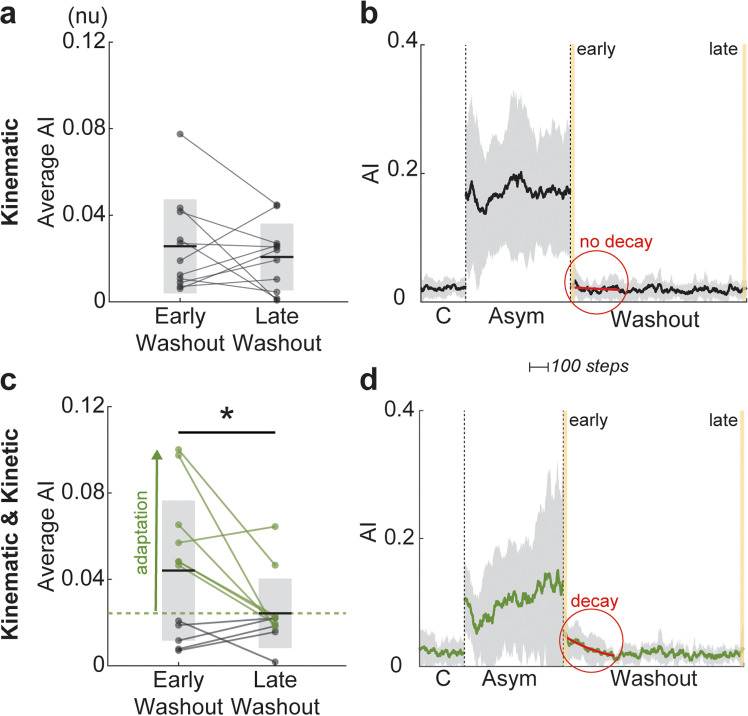
Analysis of the aftereffects in asymmetry index for Groups 1 (*kinematic* constraint only) and 2 (*kinematic* and *kinetic* constraints). (a) Comparison of the absolute value of the average AI across participants in the early (first 20 steps) and late (last 20 steps) periods of washout in Group 1. (b) The group temporal profile of AI (mean and SD) measured for each step is shown for control (C), asymmetric (Asym), and washout periods. Thin lines correspond to data for individual participants. (c-d) The same analysis is shown for Group 2 and its subgroup (green in both panels), with AI values in the early washout above the mean of the late washout (green dashed line). AI equal to 0 represents symmetric gait. * indicates a significant difference with p < 0.05. The solid red curves in (b) and (d) highlight the difference in the decay of AI between the groups.

The presence of adaptation in the form of aftereffects was tested by comparing temporal asymmetry in the early and late periods of the washout condition. Fig 4 compares the absolute value of the average AI values in the early and late portions of the washout condition. For Group 1, the data was normally distributed (early washout, p = 0.79, late washout, p = 0.89). The AI values for the early and late washout were 0.026 (±0.022) and 0.021 (±0.015), respectively, failing a paired, two-tailed t-test (p = 0.48). Thus, there was no significant difference between the early and late portions of washout with only a kinematic constraint, see Fig 4a. Thin gray lines show absolute AI values for individual participants calculated for early and late washout periods (each representing an average of 20 steps, shown in yellow in Fig 4b and 4d). The average behavior for Group 1 supports this result, see Fig 4b. In the control condition, participants were nearly symmetrical, with AI values of 0.022 (±0.002). In the asymmetric condition, the imposed asymmetry is evident with AI values of 0.171 (±0.013). In the washout condition, participants returned to near their baseline AI values of 0.019 (± 0.004) without any aftereffects from the imposed asymmetry demonstrated by a lack of exponential decay in the beginning of the washout condition (curve equation: $y=0.041e^{-0.001x}$).

For Group 2, the data was also normally distributed (early washout, p = 0.77, late washout, p = 0.07). However, unlike Group 1, the AI values for the early washout were significantly greater than those for the late washout (0.044 (±0.032) and 0.024 (±0.016)), passing a paired, right-tailed t-test (p = 0.02), see Fig 4c. Therefore, Group 2, as a whole, demonstrated de-adaptation. Moreover, there is a clear divide in participants within the group that individually showed this difference, designated by the dashed green line in Fig 4c. This line was drawn at the group average for the late washout condition, and any participant with average AI values in the early washout condition above this line were considered to have shown adaptation (N = 7) while any participant with average AI values in the early washout condition below this line were considered to not have shown adaptation (N = 5). The average behavior of the individuals within Group 2 that showed adaptation supports the results shown in Fig 4c, see Fig 4d. Like Group 1, this subset of Group 2 participants was nearly symmetrical in the control condition with AI values of 0.023 (±0.004), and the imposed asymmetry is evident in the asymmetric condition with AI values of 0.103 (±0.023). However, in the washout period, the presence of aftereffects from the imposed asymmetry is evident, indicated by the exponential asymmetry decay (curve equation: $y=1.043e^{-0.004x}$) in the beginning of the washout condition before the return to near baseline average AI values of 0.022 (±0.008). In Fig 4d, the exclusion of Group 2 participants who did not show adaptation was done to highlight the exponential decay that is present in the majority of the group, which would otherwise be masked from averaging the behavior of those with and without the decay.

## Discussion

In this study, we examined the initiation of the sensorimotor adaptation during locomotion and tested if this mechanism has an error threshold that initiates a persistent adaptation. The existence of this element is supported by observations that all people may have small and persistent asymmetric stepping that remains uncorrected. Only large limb asymmetries, for example, walking on the split-belt treadmill with 1:2 or 1:3 belt speed differences, induced persistent adaptations [21,22,38]. Thus, we expected to detect the presence of an asymmetry threshold at which human participants initiate motor adaptations. Our aim was to test whether the motor adaptation mechanism required a threshold in sensory error to trigger adaptation. This was tested by imposing a whole-limb stride limiting (kinematic) constraint using a passive orthosis or a combination of the kinematic and loading (kinetic) constraints that induced stepping asymmetry. We tested the following hypothesis: the increasing sensory interlimb asymmetry triggers a persistent locomotor asymmetry with aftereffects at high, but not low, error values. We found that a kinematic constraint alone was not sufficient and required an additional kinetic constraint to trigger persistent adaptations in most participants. The adaptation caused by limb stepping asymmetry and interlimb difference in loading resembled asymmetric limb preference.

The initiation of adaptation was, for the first time, specifically studied with the use of mild kinematic and kinetic asymmetries. The kinematic constraint was designed to mimic limb preference as in our previous animal studies [39]. Only the combination of kinematic and kinetic constraints developed a persistent adaptation with limb preference in humans walking on a tied-belt treadmill. The result of testing for the presence of adaptation after different asymmetric conditions is summarized in Fig 4. Similarly, previous studies showed that small interlimb speed differences during split-belt treadmill locomotion did not induce adaptation, while the large differences did lead to adaptation with aftereffects [40]. The increase in limb speed is positively correlated with the increase in loading, which can even be reliably used to identify limb speed in self-paced applications [41]. This would also support the expectation that large interlimb speed differences result in a large difference in interlimb loading, thus creating a kinetic imbalance.

We used an asymmetry index based on leading and trailing double-stance periods. This differs from split-belt treadmill studies [42,43], where one leg steps faster than the other, and a step-length asymmetry is imposed by the interlimb speed differences. However, the comprehensive comparison of the step-time and step-length measures pointed to the benefits of temporal asymmetry analysis for locomotor adaptation tasks, even in split-belt locomotion [44,45]. Sternum and Choi [44] showed that step-time asymmetry captured the step adaptation better than step-length asymmetry, showing less variability in this relationship and reflecting energetic cost. Step-length and step-time variables can be independently controlled, which is supported by the observation that participants can dissociate the adaptation of temporal and spatial motor outputs [46], indicating the need to examine neuromechanical dynamics during locomotor adaptation further. Here, we chose double stance as the temporal measure because it is related to the interlimb dynamics, which was associated with the robust aftereffects in the washout period, unlike the intralimb variables [14].

Why could a combination of kinematic and kinetic asymmetries, not a kinematic asymmetry alone, be the key to initiating adaptation? Our results indicate the existence of a sensory threshold for the onset of adaptation, even at small interlimb stepping asymmetries with the same interlimb speeds. When the interlimb speed difference is small, it generates turning and walking along curved paths [31,32]. This behavior falls within the domain of typical heading direction control and should not initiate a motor adaptation, as it would be disruptive to the regular turning function. It is likely that a further increase in the kinematic asymmetry may also be within the physiological repertoire of precise limb control, for example, in the context of stepping over obstacles. This logic led us to hypothesize that the kinetic asymmetry with small kinematic asymmetry would result in shifting the system to the non-standard operation regime that may require adaptation. This idea was supported by the observations of adaptations during walking with 1:2 and 1:3 belt-speed coupling. The loading in these asymmetric speed patterns is likely to be different, as can be tested with the loading analysis in our studies. Indeed, the faster limb would experience a shorter loading, thus generating an asymmetric difference in the cumulative sum of loading between limbs (see Fig 3). The increase in kinematic error may be challenging to dissociate from the accompanying kinetic error, but both signal modalities are expected to contribute to the representation of the internal dynamic state, as in the view of motor control that uses embedding of limb dynamics [19,47].

It is likely that both individual experience and morphometry may define subject-specific magnitude that determines when “on-demand” resilience to kinematic asymmetries would be insufficient. In our study, 58% of participants developed adaptation with the kinematic and kinetic constraints that promote limb preference. We expect that a further increase in the magnitude of the asymmetry condition could have resulted in adaptation. In the locomotor system, which is known to be a state-dependent dynamical system with multiple regimes [48], multiple stable states with varying inter- and intra-limb coordination may exist to accommodate variable external conditions. One extreme example is the pattern of rhythmogenic activity of isolated spinal cord, which could switch from a customary extensor-dominated to the atypical flexor-dominated pattern in response to changes in external drives [35]. Similarly, both cortical and spinal pathologies may lead to persistent locomotor adaptations or maladaptations that require further rehabilitation. Our finding of an error threshold in adaptation is novel and consistent with the typical practice of exaggerating locomotor deficits to promote locomotor rehabilitation [49,50]. The study of locomotor adaptations not only provides insight into motor learning [51], but also guides rehabilitation of symmetric walking [52,53].

Both sensorimotor adaptation and use-dependent plasticity are part of the locomotor adaptation mechanism [9,18,54]. More generally, these two mechanisms are model-based [55] and model-free [56] learning strategies utilized by the nervous system across planning and execution pathways. In our study, use-dependent learning does not explain the presence of error onset in the function responsible for the initiation of locomotor adaptation. Subjects experienced the same number of steps in the kinematic-only and kinematic-kinetic conditions. The continuous activity-dependent adjustment of sensorimotor gains for error correction would be expected to have aftereffects in both conditions. However, only the kinematic-kinetic condition led to the aftereffects in the washout period, see Fig 4. This result is similar to the observations of adaptation with large interlimb kinematic differences—1:2 or other ratios of split-belt coupling [40,57,58]—where both kinematic and kinetic interlimb asymmetries are present.

Limitations to our study pertained to the magnitude of certain parameters (e.g., walking speed, amount of step length constrained, and threshold for unloading) being consistent across participants despite individual differences in preferred walking speed, step length, and weight. Regarding walking speed, 1.0 m/s was used across all participants because some may have found a typical healthy preferred speed of 1.4 m/s to be too challenging during the imposed asymmetry period due to their morphometry and/or behavioral set. The threshold for triggering adaptation may be related to the perceived difficulty of this task; therefore, future work may normalize the walking speed during the task to the individual’s preferred speed. Similarly, within groups, the amount of constrained step length was consistent across participants due to the passive orthosis being the same distance away from the limb regardless of the individual’s typical step length. This may have also contributed to the perceived difficulty of the task and, ultimately, the threshold for triggering adaptation. Future work may normalize the configuration of the passive orthosis to the participant’s step length or height. Finally, the monitoring of the vertical ground reaction forces during the task to ensure proper unloading in the participants who experienced both kinematic and kinetic constraints was subjective to the experimenter. Future work may automate this process and normalize the threshold to be considered unloading to the participant’s weight.

Another limitation is that our study focuses on testing the evidence for the sensory threshold that initiates adaptation, but it is not designed to evaluate what types of sensory errors can be essential. Kinematic and kinetic behavioral conditions cannot isolate spatial or kinematic asymmetry in posture from underlying dynamics related to active and passive forces acting on sensors in joints and musculotendon structure. For example, the study of Blum et al. (2017) stated controversially that the muscle spindle (a muscle-indwelling sensor of muscle length and speed) is sensitive to the transient force increase [59]. Our simulations of neuromechanical systems provide insights into the interconnected nature of posture- and force-related signaling within locomotor control systems [60]. For example, the kinematic constraint on the vertical movement of the center of mass increases muscle recruitment and its force generation through the compound action of spindle and tendon organ afferents. The neuromechanical coupling of posture and load may be difficult to resolve, but specific modalities can still be preferred to initiate locomotor adaptations.

In conclusion, the presentation of sensorimotor errors can guide the modification of movement in populations with gait asymmetries due to injuries to the central nervous system, for example, stroke and spinal cord injury. This study exposes how the magnitude and modality (kinematic and kinetic) of sensory errors trigger the locomotor adaptation mechanism, which is otherwise tolerant to changes. The existence and details about the triggering process within the locomotor adaptations were not previously explored.
